# Nerve root block versus surgery (NERVES) for the treatment of radicular pain secondary to a prolapsed intervertebral disc herniation: study protocol for a multi-centre randomised controlled trial

**DOI:** 10.1186/s13063-018-2677-5

**Published:** 2018-09-05

**Authors:** Martin J. Wilby, Carolyn Hopkins, Emma Bedson, Sue Howlin, Girvan Burnside, Elizabeth J. Conroy, Dyfrig A. Hughes, Manohar Sharma, Anthony Marson, Simon R. Clark, Paula Williamson

**Affiliations:** 1 Department of Neurosurgery, The Walton Centre NHS Foundation Trust, Liverpool, L9 7LJ UK; 2 Clinical Trials Research Centre, University of Liverpool, Liverpool, L12 2AP UK; 3 Institute of Translational Medicine, University of Liverpool, Liverpool, L69 7BE UK; 4 Centre for Health Economics and Medicines Evaluation, Bangor University, Bangor, LL57 2PZ UK; 5 Department of Pain Medicine, The Walton Centre NHS Foundation Trust, Liverpool, L9 7LJ UK; 6 Department of Neurology, The Walton Centre NHS Foundation Trust, Liverpool, L9 7LJ UK

**Keywords:** Sciatica, Microdiscectomy, Transforaminal epidural steroid injection, Prolapsed intervertebral disc, Randomised controlled trial

## Abstract

**Background:**

Sciatica is a common condition reported to affect over 3% of the UK population at any time and is often caused by a prolapsed intervertebral disc (PID). Although the duration and severity of symptoms can vary, pain persisting beyond 6 weeks is unlikely to recover spontaneously and may require investigation and treatment. Currently, there is no specific care pathway for sciatica in the National Health Service (NHS), and no direct comparison exists between surgical microdiscectomy and transforaminal epidural steroid injection (TFESI). The NERVES (NErve Root block VErsus Surgery) trial aims to address this by comparing clinical and cost-effectiveness of surgical microdiscectomy and TFESI to treat sciatica secondary to a PID.

**Methods/design:**

A total of 163 patients were recruited from NHS out-patient clinics across the UK and randomised to either microdiscectomy or TFESI. Adult patients (aged 16–65 years) with sciatic pain endured for between 6 weeks and 12 months are eligible if their symptoms have not been improved by at least one form of conservative (non-operative) treatment and they are willing to provide consent. Patients will be excluded if they present with neurological deficit or have had previous surgery at the same level. The primary outcome is patient-reported disability measured using the Oswestry Disability Questionnaire (ODQ) score at 18 weeks post randomisation and secondary outcomes include disability and pain scales using numerical pain ratings, modified Roland-Morris and Core Outcome Measures Index at 12-weekly intervals, and patient satisfaction at 54 weeks. Cost-effectiveness and quality of life (QOL) will be assessed using the EQ-5D-5 L and self-report cost data at 12-weekly intervals and Hospital Episode Statistics (HES) data. Adverse event data will be collected. Analysis will follow the principle of intention-to-treat.

**Discussion:**

NERVES is the first trial to evaluate the comparative clinical and cost-effectiveness of microdiscectomy to local anaesthetic and steroid administered via TFESI. The results of this research may facilitate the development of an evidence-based treatment strategy for patients with sciatica.

**Trial registration:**

ISRCTN, ID: ISRCTN04820368. Registered on 5 June 2014.

EudraCT EudraCT2014–002751-25. Registered on 8 October 2014.

**Electronic supplementary material:**

The online version of this article (10.1186/s13063-018-2677-5) contains supplementary material, which is available to authorized users.

## Background

Sciatica is broadly defined as leg pain in the distribution of a lumbosacral nerve root [1]. It is a common condition affecting over 3% of the population at any one time and over 90% of sciatica is due to a prolapsed intervertebral disc (PID) [2]. Patients affected are typically young, working adults and it can be helpful to consider three categories of sciatica: (1) acute sciatica – lasts less than 6 weeks and may be self-limiting with little or no impact on the patient’s ability to perform usual activities; (2) chronic sciatica – persists beyond 6 weeks and has a tremendous impact upon the patient’s working ability and (3) resistant sciatica – persists beyond 12 months [3]. Although the duration of pain may vary considerably, and the natural history of sciatica is favourable within 1 year, many patients have pain that persists beyond 6 weeks which could have considerable impact upon the employment market and patients’ lives [4]. It is generally accepted that pain persisting beyond 6 weeks is unlikely to get better imminently and requires further investigation and treatment [1–4]. There is no current accepted treatment paradigm for sciatica within the UK [1]. Treatment options are largely uproven but include analgesic drugs of various categories including antiepileptics and antidepressants, injections of drug combinations into the spine and surgical techniques to remove the prolapsed disc [1] Recent evidence has suggested that the commonly used neuromodulator drug pregabalin may not have a strong benefit in the treatment of sciatica in the community [5]. Epidural steroid injections (ESI) are another treatment modality for sciatica and involve the administration of a mixture of local anaesthetic and steroid into the spine via one of three main routes; through the base of the spine (caudal epidural), through the back of the spine (inter-laminar) or through the nerve tunnel directly adjacent to the prolapsed disc (transforaminal epidural steroid injection (TFESI) [6]. Randomised controlled trials (RCTs) have looked at ESI for acute sciatica but these have not included comparisons between TFESI and inter-laminar ESI [7–10]. However, prospective and case control studies have compared these and demonstrated a superior efficacy of TFESI [6–9]. One recent study of TFESI ([9]; *n* = 238) reported that 65% of injections were effective at follow-up greater than 6 months (based on patient-reported measures) suggesting that the administration of drug closer to the disc prolapse may improve efficacy when compared to other methods of administration. Moreover, efficacy is improved if symptom duration is less than 6 months prior to injection [9]. Only one trial [10]; *n* = 100) has directly compared inter-laminar ESI to surgery for sciatica secondary to PID and suggested that ESI could prevent 50% of surgical interventions. Although this specific use of steroid is outside the marketing authorisation (off-label) it is commonly used and a widely accepted treatment for sciatica. Of the surgical techniques, microdiscectomy to remove the prolapsed disc is considered the ‘gold standard’ with reported success rates of 90% [11]. As sciatica has a good natural history there is potential that the treatment administered in the form of injection may render surgery as excessive, but results from other studies have shown that ESI only have a small short-term effect on leg pain and disability compared with placebo, and no effect in the long term [12]. These poor medium- to long-term results have given ESI poor perceived efficacy and hence they are widely ignored in the treatment of acute sciatica [13]. Perhaps because of this at the time of trial conception no care pathway in the National Health Service (NHS) suggests any particular treatment over another [1]. No direct comparison exists between surgical microdiscectomy to treat sciatica secondary to lumber disc prolapse and nerve root blocks such as TFESI. In the UK in 2010/2011 over 25,000 therapeutic ESIs were administered and over 9000 surgical procedures were performed to remove herniated lumbar disc prolapses for sciatica (HES data). The costs to the NHS in the United Kingdom (UK) are £600 per ESI and approximately £4000 for surgical microdiscectomy (which requires an average of two nights in hospital per patient) [14].

The NERVES (NErve Root block VErsus Surgery) trial is funded to compare surgical microdiscectomy to local steroid and anaesthetic administered accurately to the source of leg pain in terms against various clinical and quality of life (QOL) outcomes to determine if there should be a recommended treatment pathway for patients with sciatica secondary to a PID. Surgical microdiscectomy and TFESI will be performed as per local NHS policy. Given the cost differential between the interventions being evaluated, and the potential for differences in clinical benefit and health outcomes, an economic evaluation will be conducted alongside the trial to determine which treatment option is the best use of health-care resources.The primary objective is to compare the clinical effectiveness of TFESI and surgical microdiscectomy for sciatica secondary to PID. Secondary objectives are to compare the cost-effectiveness of TFESI and microdiscectomy for the treatment of sciatica secondary to PID and to compare QOL outcomes for both treatments.

## Methods/design

### Study design and setting

**Fig. 1:**
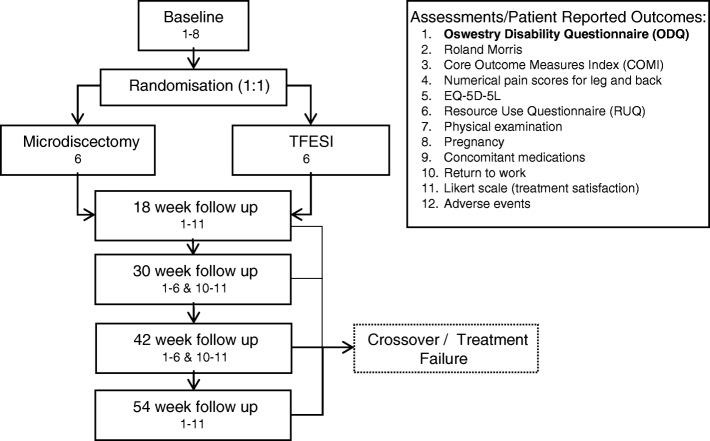
Schematic of trial design

NERVES is a two-arm, multi-centre, phase III, randomised trial comparing TFESI to surgical microdiscectomy for sciatic pain secondary to a PID. A Standard Protocol Items: Recommendations for Interventional Trials (SPIRIT) flowchart summarising the study protocol is presented in Fig. 1 (see Additional file 1 for the SPIRIT Checklist). Recruitment will occur in NHS out-patient neurosurgical, pain and orthopaedic clinics. Sites have been selected pragmatically, prior to opening a site suitability assessment including screening for the required patient volume was completed. Eligible patients who provided consent were randomised to TFESI and microdiscectomy in a ratio of 1:1 using an online computerised service. The schedule will be generated by an independent statistician, stratified by site, using permuted blocks of random sizes*.* Due to the nature of the procedures involved it was not possible to blind the participants.

Patients will be followed up for 54 weeks from the date of randomisation with follow-up visits scheduled at 18 weeks, 30 weeks, 42 weeks and 54 weeks (12-week intervals assuming treatment at 6 weeks).

Patients who have additional treatment after receiving their randomised treatment or, who do not receive their randomised treatment and instead crossover and receive the other treatment will continue with the scheduled follow-up visits, they will not be withdrawn from the trial.

**Fig. 2:**
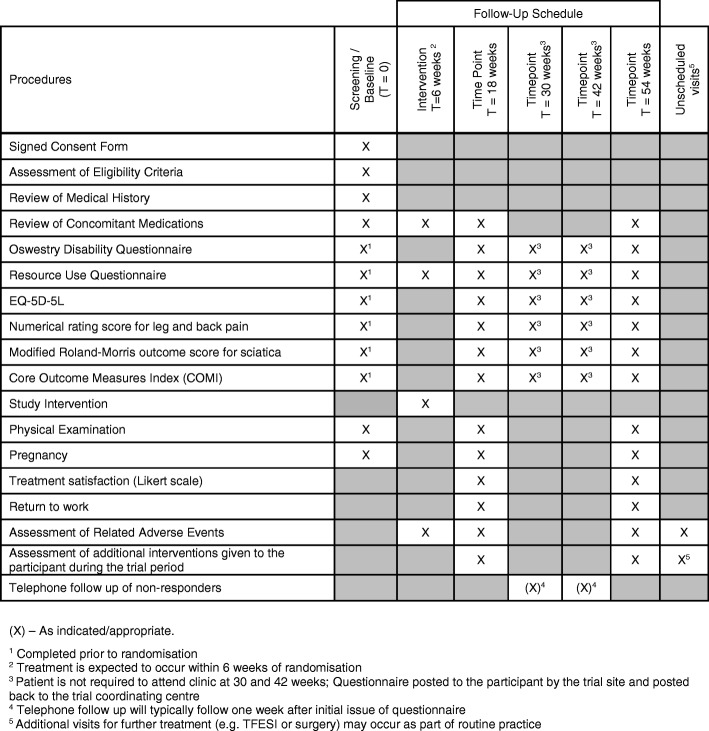
Standard Protocol Items: Recommendations for Interventional Trials (SPIRIT) Figure. Trial assessments

All participants will complete trial assessments as shown in Fig. 2.

#### Internal pilot

The original trial design includes an internal pilot phase allowing analysis of 6 months of recruitment data from two sites before progressing to full trial. The criteria for progression to full trial are:

- At least 30 patients recruited
- Consent rate of 40% or more
- Fewer than 10% of patients unhappy with allocation and receive the alternative treatment
- Fewer than 50% of patients in the injection group proceed to surgery

### Study population

The trial is open to adult patients with sciatica secondary to a PID who meet the eligibility criteria listed in Table 1. Contraindications for both arms of treatment are to be assessed on a case-by-case basis by the healthcare team as per routine NHS practice and according to local policy. Written informed consent will be obtained for all patients before any study-specific assessments are conducted. Only patients who have suffered from leg pain for more than 6 weeks of duration and who have tried at least one non-invasive treatment form were selected.

**Table 1:** Inclusion and exclusion criteria

Inclusion criteria:	
• Diagnosed lower-extremity radiculopathy (sciatica)	
• Sciatica secondary to prolapsed intervertebral disc (PID) (proven on magnetic resonance imaging (MRI))	
• Duration of symptoms between 6 weeks and 12 months	
• Leg pain non-responsive to conservative, non-invasive management	
• Age 16–65 years	
• Patient has attempted at least one form of conservative (non-operative) treatment (but this has not provided adequate relief of patient’s pain/symptoms)	
• Patient willing and able to give consent	
Including but not limited to; medication, physiotherapy, modification of daily activities	
Exclusion criteria:	
• Serious neurological deficit (e.g. foot-drop/possible cauda-equina compression)	
• Previous spinal surgery at the same intervertebral disc (level)	
• Sciatica presentation for longer than 12 months	
• Age < 16 years	
• Age > 65 years	
• Patient has not attempted any form of conservative treatment	
• Any patient who has a contraindication for surgery and/or injection	
• Patient known to be pregnant	

### Trial interventions

After baseline assessments have been completed and eligibility confirmed, patients were randomised to receive either an injection or surgery (1:1). Treatment was given within 6 weeks of randomisation where possible and should occur within 12 weeks of randomisation to ensure valid collection of primary outcome data at the 18-week follow-up. During the course of follow-up participants may require further intervention for acute sciatica as per routine NHS practice.

#### Transforaminal epidural steroid injection

TFESI is a standard nerve root blockade that will be performed as per local policy using the lateral foraminal portal of entry and guided fluoroscopically (i.e. computerised tomography (CT) or x-ray) to identify the correct level. As this is a pragmatic trial the agents used are expected to be obtained and prescribed via normal NHS routes. To minimise variability across the participating sites it is expected that the following injection regimen will be followed where possible: steroid: 20–60 mg triamcinolone, e.g. Kenalog; local anaesthetic: 0.25% levobupivacaine (2 ml), e.g. Chirocaine. Information on exact dosage and agents used, the level of injection and whether the block was preganglionic (at the level of the index disc) or postganglionic (the level below the disc) will be collected. All patients randomised to TFESI will receive at least one therapeutic injection, but may also be offered additional injections if there was partial or short-term benefit from the first injection. TFESI is an off-label use of steroid, but is commonly accepted practice within the NHS and in the wider medical field.

#### Surgical microdiscectomy

Standard microdiscectomy will be performed as per local treatment protocols. Sites will identify the correct side (left or right) and level prior to treatment with level localisation advised as per local treatment protocols. Information on site and spinal level of surgery will be collected.

### Outcomes

#### Primary outcome

The primary outcome is the Oswestry Disability Questionnaire (ODQ) at 18 weeks after randomisation (approximately 12 weeks after intervention).

#### Secondary outcomes

The secondary outcomes include the following:

1. ODQ score at 30, 42 and 54 weeks after randomisation
2. Numerical rating scores for leg and back pain at baseline, and at 18, 30, 42 and 54 weeks after randomisation
3. To assess patient treatment satisfaction at 54 weeks after randomisation
4. Modified Roland-Morris outcome score for sciatica at baseline, and at 18, 30, 42 and 54 weeks after randomisation
5. Core Outcomes Measures Index (COMI) at baseline, and at 18, 30, 42 and 54 weeks after randomisation
6. Work status (return to work and work days lost if applicable) at 18 and 54 weeks after randomisation
7. Cost-effectiveness, expressed as the incremental cost per quality-adjusted life-year (QALY) based on the EuroQol five-dimension, five-level quality of life questionnaire (EQ-5D-5 L) at baseline, and at 18, 30, 42 and 54 weeks after randomisation

### Monitoring, safety and quality control

Data will be collected using paper Case Report Forms (CRFs) and patient-completed questionnaires during the 54-week follow-up period. Data capture will be monitored in accordance with the Clinical Trials Research Centre’s standard operating procedures to ensure compliance with the International Conference on Harmonisation, Good Clinical Practice and the Research Governance Framework 2005. Adverse events (AEs) are defined by the Medicines for Human Use (Clinical Trials) Regulations 2004 (SI 2004/1031). AE data will be collected throughout follow-up; the AE reporting period begins as soon as the study intervention is received and ends 30 days after the treatment visit. Serious adverse events (SAEs) and suspected unexpected serious adverse reactions (SUSARs) will be reported as per regulatory requirements. Safety and other relevant data will be reviewed throughout the trial by an Independent Data and Safety Monitoring Committee and further oversight is provided by a Trial Steering Committee.

### Statistics

#### Statistical analysis

The ODQ is recommended as part of core outcome measures for low back research [15–18]. The scale ranges from 100 (extreme disability) to 0 (extreme ability) [16]. A change of 10 points has been widely accepted in the academic literature as the minimum clinical significance [17]. In order to detect a difference between two groups of 10 points on the ODQ at 5% significance level with 90% power, a total of 172 participants are required. This assumes a standard deviation (SD) of 20 points based on a similar population in previously published trials [13–18]. Baseline ODQ data collected on 11 potentially eligible patients from the ‘fast-track sciatica clinic’ at The Walton Centre generated an SD of 14.4, under the assumed value. We therefore initially aimed to recruit a target of 200 patients to allow for a 10% rate of missing outcome data. Randomisation was stratified by site. The primary outcome (ODQ score at 18 weeks post randomisation) will be compared between groups using a linear regression model, adjusted for the stratification variable centre, baseline ODQ score, and possibly other (specified in advance) variables considered to be potential confounders. Analysis of secondary outcomes will use similar methods, or logistic regression analyses, where appropriate. The intention-to-treat principle will be applied as far as is practically possible. The analysis set for the primary outcome will include all participants with an ODQ score at 18 weeks. Reasons for missing primary outcome data will be assessed, blind to treatment allocation, as to whether they are informative of likely outcome. Participants with non-informative reasons for missingness will be excluded from the primary analysis set. Sensitivity analyses will be carried out using multiple imputation to assess the robustness of the analysis to missing primary outcome data.

#### Revision of sample size calculation

Due to recruitment difficulties early in the trial, the sample size calculation was revisited. The original calculation did not assume any correlation between baseline and follow-up ODQ scores, as no data was available to estimate this. Based on a blinded analysis of the correlation between baseline and follow-up ODQ scores in the first 47 trial participants to have outcome data available, this correlation was estimated to be 0.49. Using this estimate, the revised sample size to achieve 90% power was found to be 66 per group. Allowing for 10% loss to follow-up gives a revised target of 74 per group (148 total). The trial was extended by the funder for a further 12 months and Steering Committees decided to continue recruitment until the end of the extension provided recruitment did not exceed 200 subjects. The trial has now stopped recruitment and 163 subjects were recruited.

#### Health economic analysis

The health economic analysis will adopt the perspective of the NHS and Personal Social Services (PSS) and additionally consider indirect costs such as time off work (secondary analysis).

Resource use associated with secondary care will be obtained from patient-level Hospital Episode Statistics (HES) data obtained from NHS Digital. Patients’ use of primary care services, personal social services, non-scheduled clinic attendance, out-of-pocket expenditures and indirect costs will be collected at baseline, treatment visit and at 18, 30, 42, 54 weeks post randomisation using a resource use questionnaire. Unit cost data will be obtained from standard sources (NHS reference costs and PSSRU Costs of Health and Social Care).

The health outcome measure will be the QALY, estimated by administering the EQ-5D-5 L at each follow-up point. The number of QALYs experienced by each patient will be calculated as the area under the curve, using the trapezoidal rule, applying the UK tariffs and corrected for baseline utility score.

Total costs will be combined with QALYs to calculate the incremental cost-utility ratio which will be compared with the £20,000 to £30,000 per QALY threshold of cost-effectiveness specified by the National Institute for Health and Care Excellence. Where appropriate, missing resource use or health outcome data will be imputed. A range of one-way sensitivity analyses will be conducted to assess the robustness of the analysis, and multivariate sensitivity analyses will be applied where interaction effects are suspected. The joint uncertainty in costs and benefits will be considered through the application of bootstrapping (10,000 replicates) and the estimation of cost-effectiveness acceptability curves. We will also employ simple parametric approaches for analysing cost and QALY data that assume normal distributions. Should the data indicate otherwise, we will develop a generalised linear model to deal with problems such as skewness.

### Dissemination of results

Study findings will be presented in conference abstracts, poster presentations and scientific publications in medical journals. The chief investigator will work with the Trial Management Group and other principal investigators to generate manuscripts for publications.

## Discussion

Surgical microdiscectomy for removal of PIDs has been shown to successfully relieve symptoms in the majority of patients [6, 11]. Disadvantages of surgery are the resource implications for the NHS due to the requirement for hospitalisation and the high skill level required of the treating physician. Surgery also carries the highest level of risk of all treatments for sciatica. Injections are relatively cheap and low risk in comparison to sciatica; they are delivered as a day-case procedure and the range of treatment providers is large, ranging from radiologists to surgeons or pain physicians. There is potential that treatment administered in the form of an injection may circumvent the need for surgery. However, the true success rate of spinal injections is largely unknown and there is no evidence comparing TFESI to surgical microdiscectomy that could be used to advise one treatment pathway over another. This protocol describes the design of a RCT to evaluate the clinical effectiveness and cost-effectiveness of TFESI to surgical microdiscectomy to treat sciatica secondary to a PID. It is the first randomised trial to address this issue to date.

### Trial status

At the time of submission, the NERVES trial was closed to recruitment. Twelve participating sites had recruited 163 patients (first patient randomised on 6 March 2015). An internal pilot had been completed at two trial sites (The Walton Centre, Liverpool, and Salford Royal Hospital) as part of an initial feasibility study and followed the same study procedures as for the main trial. The decision to progress to full trial was based on the pre-defined internal pilot criteria for progression to a full trial. No between-group statistical comparisons were carried out after the internal pilot. Only the four criteria specified for progression to the full trial were to be considered after the internal pilot.

## Additional file

Additional file 1: Standard Protocol Items: Recommendations for Interventional Trials (SPIRIT) Checklist. (DOC 127 kb)

## Abbreviations

AE: Adverse event
COMI: Core Outcomes Measures Index
CRF: Case Report Form
CT: Computerised tomography
ESI: Epidural steroid injection
HES: Hospital Episode Statistics
MRI: Magnetic resonance imagery
NHS: National Health Service
NIHR: National Institute of Health Research
ODQ: Oswestry Disability Questionnaire
PID: Prolapsed intervertebral disc
QALY: Quality-adjusted life-year
QOL: Quality of life
SAE: Serious adverse event
TFESI: Transforaminal epidural steroid injection
